# Letter to the Editor: Promising therapeutic targets for ischemic stroke identified from plasma and cerebrospinal fluid proteomes: a multicenter Mendelian randomization study’

**DOI:** 10.1097/JS9.0000000000001430

**Published:** 2024-04-04

**Authors:** Mengge Gao, Xujia Li, Lingli Huang

**Affiliations:** a Department of Clinical Nutrition, Huadu District People’s Hospital, Huadu; b VIP Department, Sun Yat-sen University Cancer Center; c State Key Laboratory of Oncology in South China, Sun Yat-sen University Cancer Center; d Collaborative Innovation Center for Cancer Medicine, Sun Yat-sen University Cancer Center; e Guangdong Provincial Clinical Research Center for Cancer, Sun Yat-sen University Cancer Center, Guangzhou, People’s Republic of China

*Dear Editor,*

I am writing to address some concerns regarding the proposition of Lanadelumab as a potential treatment for ischemic stroke (IS), as discussed in the recent study by Zou *et al*.^1^. While I appreciate the innovative approach taken by them in exploring the relationship between prokinetic prekallikrein and IS and the potential of Lanadelumab as a therapeutic agent, I believe there are several important considerations that merit attention.

Firstly, I noted the findings of another Mendelian randomization (MR) study, which presented a different perspective, indicating a positive association between genetically predicted high plasma prekallikrein concentrations and an increased risk of various stroke subtypes, including ischemic stroke^2^. This discrepancy suggested a complex role for prekallikrein in stroke development, necessitating further investigation to elucidate its precise impact and potential as a therapeutic target.

Secondly, mechanistically, Lanadelumab works by binding to an enzyme within the plasma called kallikrein and inhibiting its activity^3^. Kallikrein is a protease that plays a crucial role in the blood clotting cascade and inflammation processes^4^. One of its important functions is to cleave kininogen, a precursor protein, to generate kinins, including bradykinin, which is a potent vasodilator^5^. However, the protective effect of prekallikrein against ischemic stroke, as observed by Zou *et al*.^1^, raises a pertinent concern. The reduction of kallikrein levels by Lanadelumab could theoretically exacerbate thrombotic risk, potentially contradicting its intended therapeutic effect. This paradox warrants thorough investigation to determine the net effect of Lanadelumab on ischemic stroke incidence and progression.

Moreover, to comprehensively evaluate the impact of Lanadelumab on ischemic stroke, additional research avenues should be pursued. Long-term follow-up studies assessing the incidence of ischemic stroke in Lanadelumab-treated populations, coupled with animal model investigations into the drug’s effects on thrombosis and vascular function, are warranted. Furthermore, considerations such as dosage, treatment duration, and patient characteristics should be factored into study designs to provide a nuanced understanding of Lanadelumab’s therapeutic potential in IS.

In conclusion, while the study by Zou *et al*. contributes valuable insights into the relationship between prekallikrein and ischemic stroke, caution is warranted in extrapolating these findings to advocate for Lanadelumab as a viable treatment option. Continued research efforts are needed to elucidate the complex mechanisms underlying IS pathogenesis and to determine the suitability of Lanadelumab as a therapeutic intervention.

## Ethical approval

Not applicable.

## Consent

Not applicable.

## Sources of funding

This study was supported by Guangzhou Huadu District People’s Hospital research fund project (grant number: C202401).

## Author contribution

M.G.: conception and writing; L.H. and X.L.: conceptualization, visualization, and supervision.

## Conflicts of interest disclosure

The authors declare that they have no conflicts of interest.

## Research registration unique identifying number (UIN)

Not applicable.

## Guarantor

Dr Lingli Huang.

## Data availability statement

This correspondence relies solely on publicly available internet resources, cited in the ‘References’ section. No new data were generated or reported here, so no datasets are available for deposit in any academic repository.

## Provenance and peer review

Not commissioned, externally peer-reviewed.

## References

[1] Zou X Wang L Wang S . Promising therapeutic targets for ischemic stroke identified from plasma and cerebrospinal fluid proteomes: a multicenter Mendelian randomization study. Int J Surg 2024;110:766–776. 38016292 10.1097/JS9.0000000000000922 PMC10871597

[2] Wang Y Jia Y Xu Q . Association between prekallikrein and stroke: a Mendelian randomization study. J Am Heart Assoc 2023;12:e030525. 37581399 10.1161/JAHA.123.030525 PMC10492928

[3] Syed YY . Lanadelumab: a review in hereditary angioedema. Drugs 2019;79:1777–1784. 31560114 10.1007/s40265-019-01206-w

[4] Bekassy Z Lopatko Fagerström I Bader M . Crosstalk between the renin–angiotensin, complement and kallikrein–kinin systems in inflammation. Nat Rev Immunol 2022;22:411–428. 34759348 10.1038/s41577-021-00634-8 PMC8579187

[5] Banerji A Bernstein JA Johnston DT . Long-term prevention of hereditary angioedema attacks with lanadelumab: the HELP OLE Study. Allergy 2022;77:979–990. 34287942 10.1111/all.15011 PMC9292251

